# Co-administration of an effector antibody enhances the half-life and therapeutic potential of RNA-encoded nanobodies

**DOI:** 10.1038/s41598-023-41092-7

**Published:** 2023-09-05

**Authors:** Moritz Thran, Marion Pönisch, Hillary Danz, Nigel Horscroft, Konstantin Ichtchenko, Saul Tzipori, Charles B. Shoemaker

**Affiliations:** 1 CureVac SE, 72076 Tübingen, Germany; 2 Department of Infectious Disease and Global Health, Tufts Cummings School of Veterinary Medicine, North Grafton, MA 01536 USA; 3 Department of Biochemistry and Molecular Pharmacology, New York University School of Medicine, New York, NY 10016 USA

**Keywords:** Biotechnology, Drug discovery, Immunology, Molecular biology, Diseases, Molecular medicine

## Abstract

The incidence of *Clostridioides difficile* infection (CDI) and associated mortality have increased rapidly worldwide in recent years. Therefore, it is critical to develop new therapies for CDI. Here we report on the development of mRNA-LNPs encoding camelid-derived V_H_H-based neutralizing agents (VNAs) targeting toxins A and/or B of *C. difficile*. In preclinical models, intravenous administration of the mRNA-LNPs provided serum VNA levels sufficient to confer protection of mice against severe disease progression following toxin challenge. Furthermore, we employed an mRNA-LNP encoded effector antibody, a molecular tool designed to specifically bind an epitopic tag linked to the VNAs, to prolong VNA serum half-life. Co-administration of VNA-encoding mRNA-LNPs and an effector antibody, either provided as recombinant protein or encoded by mRNA-LNP, increased serum VNA half-life in mice and in gnotobiotic piglets. Prolonged serum half-life was associated with higher concentrations of serum VNA and enhanced prophylactic protection of mice in challenge models.

## Introduction

As one of the leading causes of nosocomial infection, *Clostridioides difficile* (formerly *Clostridium difficile*) infection (CDI) is associated with various clinical outcomes, from mild symptoms to life-threatening colitis and diarrhea^1, 2^. The Centers for Disease Control and Prevention (CDC) mentioned an incidence of approximately 200,000 infections and almost 13,000 fatalities in the US per year in 2019^3^. The main virulence factors of *C. difficile*, an anaerobic, Gram-positive bacterium, are two toxins, termed TcdA and TcdB^4^. Previously, we reported on the development of a V_H_H heterotetrameric antitoxin (VNA2-Tcd), consisting of V_H_Hs from two bispecific V_H_H-based neutralizing agents (VNAs) that target TcdA or TcdB, which was highly efficacious in various animal models of CDI^5^.

V_H_Hs, also called nanobodies, are single chain antibody fragments, derived from the V_H_ regions of heavy-chain-only antibodies (Abs) found in animals of the Camelidae family. V_H_Hs display excellent binding properties comparable to single-chain Fv (scFv) domains which derive from conventional Abs. In addition, as small, tightly compacted, disulfide-stabilized proteins, V_H_H are typically better expressed in recombinant hosts^6^ and are more resistant to extremes of temperature or pH than mAbs^7, 8^. These well-folded single domain V_H_H generally remain functional when produced as homo- or hetero-multimers. Multimeric V_H_Hs have frequently been shown to have substantially improved affinities and potencies in therapeutic applications compared to monomeric V_H_H^9–18^.

One potential drawback of V_H_Hs as therapeutics is their lack of an antibody Fc domain to promote much longer serum half-life, and to provide other antibody effector functions such as promoting opsonization of pathogens, Ab-dependent cytotoxicity or complement activation. To overcome such limitations, fusions to host-specific Fc regions or other serum abundant proteins such as albumins allow improved serum persistence and enable additional functionalities^19–24^. However, expression of these complex recombinant fusion proteins generally requires more costly eukaryotic host systems to facilitate proper protein folding and to include carbohydrate modifications^25^.

Recent advances in the development of gene therapies allow circumventing the need for ex vivo production of recombinant proteins. Among many gene therapy vectors, adeno, adeno-associated and lentiviral vectors represent the most frequently used technologies (reviewed in^26^). Viral gene therapies rely on sustained protein production offering great promise for long-term treatments. However, sustained protein expression over longer periods of time is not desired for some indications. As an alternative, treatment with formulated messenger RNA (mRNA) allows a transient defined burst of protein production and has recently proven its potential as a safe and effective therapeutic in the form of SARS-CoV-2 vaccines^27^.

Among the various current technological advancements^28^, lipid nanoparticles (LNPs) remain the gold standard to formulate and deliver messenger RNA (herein referred to as mRNA-LNPs) due to various aspects. From a physicochemical perspective, formulating mRNAs into mRNA-LNPs is a well characterized and controlled process^29^. It enables high drug product concentrations and encapsulation efficiencies as well as the potential for upscaling and mass production. This approach was amply validated by the rapid availability of billions of mRNA-LNPs doses against SARS-CoV2.

Besides vaccine applications, mRNA is being investigated for delivery of a wide variety of protein therapeutics. As examples, this strategy is showing promise for gene editing^30^, delivery of mAb products^31, 32^ and treating rare diseases with high medical need^33–36^.

A general feature of mRNA-LNPs is the tropism for hepatic delivery and the rapid onset of protein expression^37^. Upon intravenous administration, host hepatocytes immediately become the main source of protein production, enabling a means for passive vaccination to treat or prevent viral infections, tumor progression and intoxication (reviewed in^38, 39^).

The concept of in vivo production provides a plethora of possibilities for sequence engineering, and as such, also allows the design of complex agents. For example, LNP formulated mRNA-encoded multimeric V_H_H-based neutralizing agents (VNAs) fused to an albumin binding peptide (ABP) retained a moderate serum half-life and proved efficacious in vivo^37^. Instead of engineering new fusion proteins with additional functionalities, VNAs can be employed in a two-component system containing a tagged therapeutic VNA and a tag-binding antibody ‘effector’ component. An anti-tag effector antibody (EfAb) has been previously shown to provide Fc effector functions that enhance the efficacy of target-binding VNAs in such a two-component system^13, 14^.

In this report, we test the efficacy of mRNA-LNPs encoding multivalent VNAs targeting *Clostridioides difficile* toxins TcdA and/or TcdB. We show that the VNAs protect mice from the pathology of TcdA and/or TcdB exposure. Furthermore, we employ a previously identified anti-tag mAb as an EfAb^40^ to promote prolonged serum half-life to expand the window of prophylactic antitoxin efficacy of the Tcd-targeting VNAs. In immunocompetent mice, we found that co-administration of a recombinant EfAb or an mRNA-LNP-encoded EfAb led to a multifold increase in the effective VNA half-lives. A final evaluation of the two-component system (mRNA-LNP encoding both VNA and EfAb) was performed in gnotobiotic piglets which provided more robust quantification of the EfAb-enhanced VNA levels and serum half-lives. In summary, these studies confirm the potential of the mRNA-encoded two-component VNA/EfAb therapeutic strategy, such as for antitoxin prophylaxis of CDI pathology.

## Results

### Expression and characterization of mRNA encoded Tcd-neutralizing V_H_H heteromultimeric VNAs

**Figure 1:**
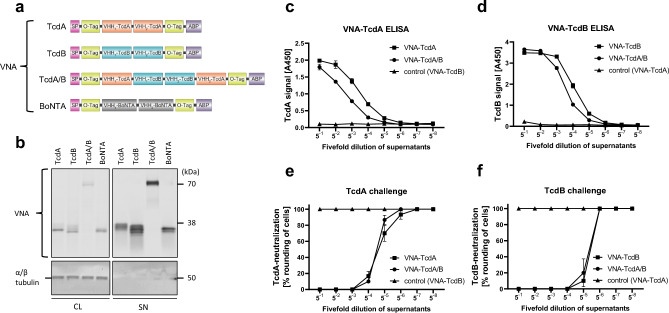
In vitro characterization of mRNA-encoded VNAs. (**a**) Design of VNA targeting *C. difficile* toxins (Tcd) A and/or B, *C. botulinum* toxin (BoNT) A, including a signal peptide (SP), V_H_H domains specific for the toxins, O-tag epitopes (O-tag), and an albumin binding peptide (ABP). (**b**) Western blot analysis of VNA-TcdA, VNA-TcdB, VNA-TcdA/B and VNA-BoNTA from cell lysates (CL) and supernatants (SN) of transfected BHK cells harvested 48 h after transfection. Cells were transfected in triplicates, and for each VNA, a representative sample was loaded on denaturing SDS-PAGE. Signals correspond to individual VNAs (upper blot) and α/β tubulin (lower blot) as a loading control. An uncropped version can be found in Supplement Fig. 11. (**c**) Detection of VNA-TcdA and VNA-TcdA/B from BHK supernatants (conditioned medium) by O-tag detection ELISA (Supplement Table 2). Control cells were transfected by VNA-TcdB. TcdA toxin was used as capture agent. Supernatants from (**b**) were assayed in 96 well-plates, starting at a 1:5 dilution (5^−1^) and serially diluted in a fivefold dilution series. A450 values (y-axis) are plotted against the dilution (x-axis). Error bars represent the standard deviation (SD) from triplicates. (**d**) Detection of VNA-TcdB and VNA-TcdA/B from BHK supernatants (conditioned medium) or medium-only (control) by O-tag detection ELISA (Supplement Table 2). Control cells were transfected by VNA-TcdA. TcdB toxin was used as capture agent. Supernatants from (**b**) were assayed in 96 well-plates, starting at a 1:5 dilution (5^−1^) and serially diluted in a fivefold dilution series. A450 values (y-axis) are plotted against the dilution (x-axis). Error bars represent the SD from triplicates. (**e**) Neutralization assay to analyze VNA-TcdA and VNA-TcdA/B functionality. Serial dilutions from c) were subjected to a neutralization assay in Vero cells. Error bars represent the SD from triplicates. (**f**) Neutralization assay to analyze VNA-TcdB and VNA-TcdA/B functionality. Serial dilutions from d) were subjected to a neutralization assay in Vero cells. Error bars represent the SD from triplicates.

Previously, we reported on the characterization and therapeutic potential of mRNA encoded VNAs targeting *C. botulinum* toxin A (VNA-BoNTA)^37^.To encode VNAs targeting the toxins of *C. difficile* (TcdA and TcdB), two V_H_Hs targeting non-overlapping toxin epitopes were incorporated in a similar framework as reported before (VNA-TcdA and VNA-TcdB, Fig. 1a). In addition, a heterotetrameric protein containing four V_H_Hs from VNA-TcdA and VNA-TcdB^5^ (VNA-TcdA/B, Fig. 1a) was encoded by mRNA. Each VNA was designed to contain a signal peptide (SP), two copies of an O-tag epitope and a carboxyl terminal murine albumin binding peptide (ABP) that promotes longer serum stability in mice^41^ (Fig. 1a). To characterize the mRNA constructs and their encoded products, baby hamster kidney (BHK) cells were transfected and the expressed proteins analyzed by western blot (Fig. 1b). Proteins of expected size were detected in both cell lysates and supernatants (Fig. 1b). Additionally, subjecting those supernatants to ELISAs specific for the individual VNAs (O-tag detection ELISA, Supplement Table 2) in a fivefold dilution series confirmed their target binding specificities (Fig. 1c,d). Finally, the function of the secreted VNAs was confirmed by their ability to prevent the toxin-induced rounding of Vero cells in neutralization assays using either toxin (Fig. 1e,f).

**Figure 2:**
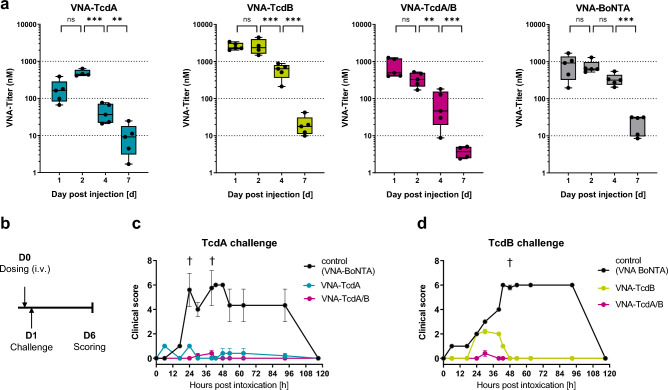
In vivo characterization of mRNA-LNP-encoded VNAs. (**a**) Quantification of serum VNA levels by O-tag detection ELISA (Supplement Table 2) at various time points. Outbred CD-1 mice received a single intravenous injection of 10 µg mRNA-LNP encoding respective VNAs. Data are depicted as whisker plots showing min to max values. For each timepoint, an independent cohort of five mice was used. *p < 0.05, **p < 0.01, ***p < 0.001. (**b**) Illustration of dosing and challenge regimen. Outbred CD-1 mice received a single intravenous injection of 10 µg of mRNA-LNP encoding individual VNAs. After 24 h, animals were challenged with 25 ng of either TcdA or TcdB toxin. Following challenge, animals were scored for clinical symptoms for a period of 120 h (day 6 post injection). (**c**) Protective efficacy of mRNA-LNP encoded VNAs in mice exposed to TcdA challenge according to schedule illustrated in (**b**). Mice which received 10 µg of mRNA-LNP encoding either VNA-TcdA (blue), VNA-TcdA/B (pink) or VNA-BoNTA (grey, irrelevant VNA) were scored for symptoms of toxemia (Supplement Table 3) and euthanized when exceeding the humane endpoint. Error bars represent the standard error (SEM) of five mice per groups. †Individual animals which succumbed to toxin challenge (group indicated by color). (**d**) Protective efficacy of mRNA-LNP encoded VNAs in mice exposed to TcdB challenge according to schedule illustrated in (**b**). Mice which received 10 µg of mRNA-LNP encoding either VNA-TcdB (green), VNA-TcdA/B (pink) or VNA-BoNTA (grey, irrelevant VNA) were scored for symptoms of toxemia (Supplement Table 3) and euthanized when exceeding the humane endpoint. Error bars represent the SEM of five mice per groups. †Individual animals which succumbed to toxin challenge (group indicated by color).

Following in vitro characterization, we evaluated whether VNAs are detectable and functional in vivo. To this end, VNAencoding mRNAs were formulated as mRNA-LNPs and administered intravenously to outbred CD-1 mice in several pharmacokinetic (PK), dose finding, and challenge studies. To estimate the expression kinetics of Tcd-neutralizing V_H_H heteromultimeric VNAs, mice received a single intravenous injection of 10 µg of mRNA-LNP encoding VNAs and serum was prepared at different timepoints following injection. VNAs were quantified by ELISAs specific for the individual VNAs (O-tag detection ELISA, Supplement Table 2) in a fivefold dilution series using recombinant proteins for quantification (Fig. 2a). Among the tested constructs, VNA-TcdB expressed at the highest levels. Serum levels of all VNAs encoded by mRNA-LNPs peaked within the first two days following administration, declined through day 4 and were only marginally detectable at day 7.

Functionality of in vivo expressed VNAs was assessed in a toxin challenge experiment. 24 h following a single intravenous injection of 10 µg of VNA encoding mRNA-LNPs, mice were challenged with 25 ng of either TcdA or TcdB toxin, previously shown to be a lethal dose^17^. Animals were scored for clinical symptoms associated with toxemia (scoring according to Supplement Table 3) for a period of five days following challenge (illustrated in Fig. 2b). Animals which had received mRNA-LNP encoding VNAs corresponding to the administered toxin (TcdA or TcdB) developed mild or moderate clinical symptoms that typically resolved within 2 days. All animals which had received mRNA-LNP encoding the irrelevant VNA-BoNTA developed severe symptoms of toxemia upon challenge with either toxin. Despite severe symptoms, 3/5 of these mice unexpectedly survived TcdA challenge and 4/5 survived the TcdB challenge and recovered after 5 days. Employing quantitative body clinical scores in place of lethality (Fig. 2c,d), the improved outcomes clearly showed that the mRNA-LNP encoded VNAs retained in vivo antitoxin function.

### Characterization of an mRNA encoded affinity tag binding effector antibody

The serum half-life of VNAs in mice is only 1–2 h which can be extended to about a day by including an ABP^37^. To further improve serum VNA half-life and add Fc effector functions, we employed a previously developed high affinity rat anti-O-tag mAb^40^ as an EfAb that binds to O-tag epitopes present on the VNAs. To produce this mAb by mRNA, we encoded the rat mAb V_H_ and V_L_ joined to a human IgG1 framework known to produce significant antibody levels both in vitro and in vivo^37^. As a benchmark, the rat hybridoma IgG1 was included in several in vivo studies as recombinant protein. For initial characterization of the mRNA-encoded EfAb (referred to as RNA-EfAb), mRNAs encoding both, heavy chain (HC) and light chain (LC) of the EfAb were mixed at a 1.5:1 molar ratio (HC:LC) and transfected into BHK cells. For comparison, a previously described anti-rabies antibody was transfected (RNA-SO57^37, 42^,). After 48 h, cell lysates and supernatants were analyzed by western blot to confirm the expression and secretion of heavy and light chains (Supplement Fig. 1a). To optimize protein expression, different ratios of HC- and LC-encoding mRNAs were transfected into BHK cells and protein levels were quantified after 24 h by IgG specific ELISA. In line with previous reports^37^, expression was highest using equimolar ratio (molar ratio of 1:1; HC:LC) or heavy chain in excess (molar ratio of 1.5:1; HC:LC; Supplement Fig. 1b). For studies reported here, a molar ratio of 1.2:1 (HC:LC) co-formulated into mRNA-LNPs was used.

**Figure 3:**
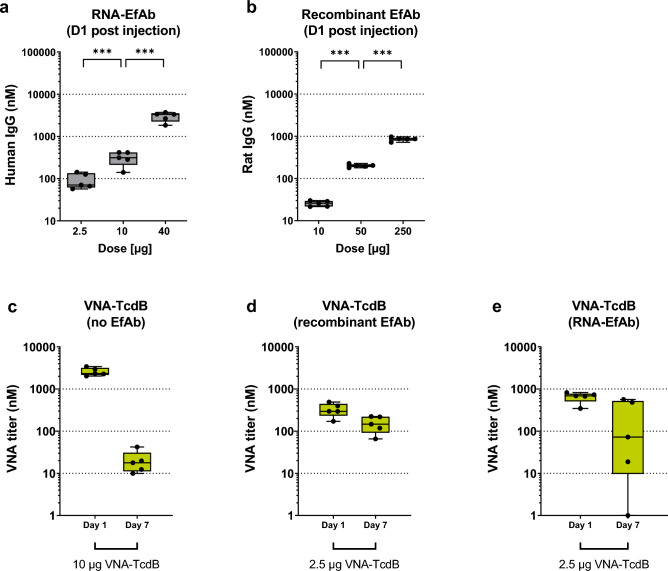
Co-administration of EfAb enhances the half-life of VNAs in vivo. (**a**, **b**) Quantification of serum titer, one day following a single intravenous injection of various doses of mRNA-LNP encoding EfAb (**a**, Total human IgG ELISA, Supplement Table 2) or recombinant rat EfAb (**b**, rat EfAb ELISA, Supplement Table 2) in outbred CD-1 mice. Data is depicted as whisker plots showing min to max values of five individual mice. *p < 0.05, **p < 0.01, ***p < 0.001. (**c**) Quantification of serum VNA-TcdB titer (V_H_H detection ELISA, Supplement Table 2) at either Day 1 or Day 7 following a single intravenous injection of 10 µg of mRNA-LNP encoding VNA-TcdB in outbred CD-1 mice (same groups as shown in Fig. 2a). Data is depicted as whisker plots showing min to max values. For each timepoint, an independent cohort of five mice was used. *p < 0.05, **p < 0.01, ***p < 0.001. (**d**, **e**) Quantification of serum VNA-TcdB titer (V_H_H detection ELISA, Supplement Table 2) at either Day 1 or Day 7 following a single intravenous injection of 2.5 µg of mRNA-LNP encoding VNA-TcdB together with either 100 µg of co-administered recombinant rat EfAb (**d**) or 10 µg of mRNA-LNP encoded human EfAb (**e**) in CD-1 mice. Data is depicted as whisker plots showing min to max values. For each timepoint, an independent cohort of five mice was used.

To establish EfAb mRNA doses for co-administration with VNAs, dose–response studies were separately performed with each of the VNA- and EfAb-encoding mRNAs and the recombinant rat EfAb protein (Fig. 3a,b). Like in prior studies^37^, mRNA-LNP encoding EfAb was dosed in a range between 2.5 µg to 40 µg, whereas recombinant EfAb was dosed in a range between 10 µg to 250 µg. The VNA and EfAb serum levels were analyzed 24 h after intravenous injection into mice. As seen previously^37^, the RNA-encoded EfAb produced a superlinear curve with each fourfold increase in RNA dose resulting in a somewhat greater than fourfold increase in EfAb production (Fig. 3a). In contrast, the recombinant EfAb serum levels closely reflected the administered doses (Fig. 3b). A similar superlinear serum VNA dose response was observed 24 h post administration of mRNA-LNPs encoding the VNAs (Supplement Fig. 2a-c). Interestingly, this superlinear dose response after intravenous injection has not been observed in non-human primates (Supplement Fig. 10a).

Based on these results we estimated that 10 µg of mRNA-LNP encoding EfAb or 100 µg of recombinant EfAb result in serum EfAb levels sufficient to bind all O-tags present on the serum VNAs following coadministration with 2.5 µg of mRNA-LNPs encoding a VNA.

### Co-administration of EfAb extends serum survival of tagged VNAs

We next tested the impact of co-administered EfAb on serum VNA half-life in mice. Once EfAbs occupy the VNA O-tags, the serum VNAs cannot be detected employing anti-O-tag reagents. Thus, to enable the detection of total serum VNA in the presence of co-administered EfAb, an ELISA specific for V_H_H domains was employed (V_H_H detection ELISA; Supplement Table S2). VNA-TcdB encoding mRNA-LNPs (2.5 µg) were co-administered together with either 100 µg of recombinant or 10 µg of mRNA-LNP encoded EfAb into mice and serum was prepared. VNA-TcdB levels when co-administered with EfAb remained at approximately 50 percent of their peak levels at day 7 of the experiment (Fig. 3d). In the absence of EfAb, levels of VNA-TcdB declined to approximately one percent of their initial peak levels (Figs. 3c). The prolonged serum half-life of co-administered EfAb of VNA-TcdB was not observed in these samples using anti-O-tag reagents for detection, clearly showing that, after 7 days, only a small fraction of the two O-tags on each VNA in the serum were uncomplexed to EfAbs (Supplement Fig. 3). This observation strongly suggests that it is only the VNAs complexed to EfAb that acquire prolonged serum persistence.

Upon co-administration of mRNA-LNP encoded humanized EfAb, only a subset of mice displayed the same persistent serum VNA-stabilizing influence observed using the rat recombinant EfAb (Fig. 3d, e). This heterogenous outcome was observed in various cohorts of mice harvested and analyzed at different timepoints (Supplement Fig. 4a). We hypothesized that the heterogeneous outcome in the immunocompetent outbred mice results from induction of anti-drug antibodies (ADA). Similar findings have been previously observed for some RNA-encoded antibodies^37^.

To investigate potential ADA, we tested the mouse sera using ADA-specific ELISAs (Supplement Figs. 5e and 6e). Due to the lack of standards for ADA quantification, the OD values (A450 signals) obtained from fivefold dilutions series were assigned a score (Supplement Fig. 5a,b) defined as ‘the greatest serum dilution resulting in a signal above background’. Some serum samples obtained at the later timepoints following administration displayed clearly elevated VNA ADA scores (Supplement Fig. 5a,b). To correlate the ADA scores with the observed VNA PK profile, a comparable scoring was performed on the VNA-TcdB signals (Supplement Fig. 5c,d). Correlation of VNA scores and VNA ADA scores confirmed that ADA positive sera contained low VNA levels (Supplement Fig. 4b). Moreover, we investigated if the serum EfAb levels were also affected by an ADA response (Supplement Fig. 6). As with VNA levels, EfAb levels also were highly variable at later time points (Supplement Fig. 6a–d). Following the same scoring logic, we again observed a strong association between serum with low EfAb levels and high EfAb ADA scores (Supplement Fig. 4c). A final correlation analysis revealed that animals which had developed ADA against the VNA also displayed ADA against the EfAb (Supplement Fig. 4d). This analysis confirmed that the serum abundance of VNA-TcdB in some animals was impacted by ADA responses to both the VNA and the EfAb. Taken together, our findings suggest that in general the co-administration of EfAb can significantly enhance the serum persistence of O-tagged VNAs in mice. The results also demonstrated that, without carefully de-immunizing both the VNA and the EfAb, ADA responses to both VNA and EfAb can rapidly develop in some mice about a week post administration that severely diminish the serum half-life of these agents.

### Co-administration of EfAb extends the protective efficacy of VNAs

**Figure 4:**
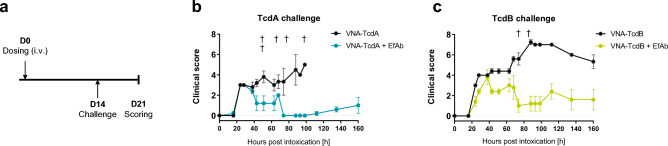
Co-administration of mRNA-LNP encoded EfAb prolongs the protective activity of VNAs. (**a**) Illustration of dosing and challenge regimen. Outbred CD-1 mice received a single intravenous injection of mRNA-LNP. After 14 days, animals were challenged with 50 ng of either TcdA or TcdB toxin. Following challenge, animals were scored for clinical symptoms for a period of 160 h (day 21 post injection). (**b**) Long-term protective efficacy of mRNA-LNP-encoded VNAs and co-administered EfAb in mice exposed to TcdA challenge according to schedule illustrated in (**a**). Mice which received 2.5 µg of mRNA-LNP encoding VNA-TcdA either alone (grey) or together with 10 µg of mRNA-LNP encoding EfAb (blue) were scored for symptoms of toxemia (Supplement Table 3) and euthanized when exceeding the humane endpoint. Error bars represent the SEM of five mice per group. †Individual animals which succumbed to toxin challenge (group indicated by color). (**c**) Long-term protective efficacy of mRNA-LNP-encoded VNAs and co-administered EfAb in mice exposed to TcdB challenge according to schedule illustrated in a). Mice which received 2.5 µg of mRNA-LNP encoding VNA-TcdB either alone (grey) or together with 10 µg of mRNA-LNP encoding EfAb (green) were scored for symptoms of toxemia (Supplement Table 3) and euthanized when exceeding the humane endpoint. Error bars represent the SEM of five mice per group. †Individual animals which succumbed to toxin challenge (group indicated by color).

Since the co-administration of EfAb extended the serum half-life of mRNA-LNP encoded VNAs, we next sought to establish that the therapeutic window for prevention of severe intoxication was similarly prolonged in mice. To this end, mRNA-LNPs encoding VNA-TcdA or VNA-TcdB were administered in the presence or absence of mRNA-LNP encoding EfAb. Two weeks following RNA administration, mice were challenged with 50 ng of either TcdA or TcdB toxin and their clinical scores were monitored for the next six to seven days (illustrated in Fig. 4a; scoring based on Supplement Table 3). To increase lethality, a twofold higher toxin dose was chosen compared to the initial challenge study (Fig. 2c,d). On average, mice that received mRNA-LNP encoding VNAs but not the EfAb showed much more severe signs of intoxication than mice which had also received the mRNA-LNP encoding EfAb (Fig. 4b,c).While mice that received EfAb had relatively mild symptoms and suffered no deaths following toxin exposures, all mice lacking EfAb died upon TcdA challenge (5/5) and following TcdB challenge, all mice developed more severe clinical symptoms and two died (2/5).

Compared to the previous challenge study (Fig. 2c,d), protection in mice which received mRNA-LNP encoding both, VNA and EfAb was less robust, particularly for the VNA-TcdB challenge. We suspected this was again due to variable occurrence of ADA as demonstrated before. We thus assessed total VNA-TcdB and EfAb levels and ADA against either protein at the time of the challenge (Supplement Fig. 7b–f). Among five treated animals, both individuals with severe clinical outcome had no detectable VNA or EfAb levels and displayed clear signs of ADA. Taken together, in the absence of ADA, EfAb co-expression clearly enhanced the therapeutic window for protection against severe intoxication.

### Co-treatments of piglets with anti-tag EfAb improves serum levels and half-life of tagged VNAs

**Figure 5:**
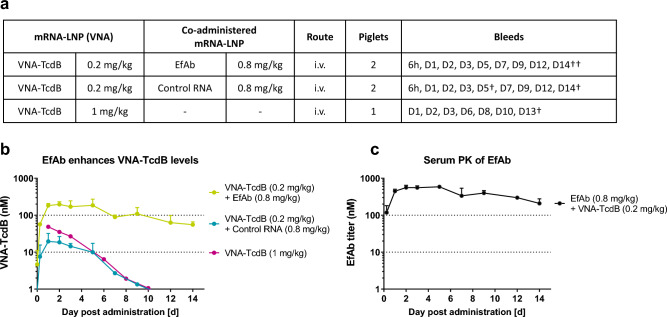
Co-expression of EfAb promotes increased levels and enhances the half-life of serum VNA-TcdB. (**a**) Table summarizing the dosing and sampling schedule for gnotobiotic piglets in (**b**) and (**c**). †Termination of individual animals. In group 2, following Day 5, only a single animal remained in the study. (**b**) Quantification of VNA-TcdB levels in piglets (V_H_H detection ELISA) following a single intravenous injection of mRNA-LNP encoding VNA-TcdB, either together with mRNA-LNP encoding EfAb (depicted in Fig. 5c; n = 2) or a control mRNA-LNP (n = 2; n = 1 following day 5, control mRNA encoded a non-translated GFP) or VNA-TcdB alone (n = 1). Serum VNA-TcdB values were determined by V_H_H detection ELISA (Supplement Table 2). Data is depicted as mean. Error bars represent the SD of two replicates. (**c**) PK profile of co-administered EfAb in piglets which had received. a single intravenous injection of mRNA-LNP encoding EfAb together with mRNA-LNP encoding VNA-TcdB (depicted as lime group in Fig. 5b) were determined using Total human IgG ELISA (Supplement Table 3). Data is depicted as mean. Error bars represent the SD of two replicates.

To test the influence of EfAb on the serum levels and the half-life of tagged VNA-TcdB in a larger animal model more amenable for human enteric disease studies, a PK study was performed in gnotobiotic piglets. Employing piglets provided both, a non-rodent model, and the feasibility to obtain significant quantities of serum from the same animals at multiple time points. Furthermore, since in piglets the murine ABP component present on the mRNA encoded VNAs does not bind porcine albumin (Supplement Fig. 8a), the serum half-life of VNAs expressed in the absence of EfAb reflects the intrinsic PK of mRNA-LNP encoded VNAs (Supplement Fig. 8b). Piglets were administered 0.2 mg/kg of mRNA-LNP encoding VNA-TcdB and co-injected with 0.8 mg/kg of either mRNA-LNP encoding EfAb or a control mRNA-LNP encoding a non-translated GFP. All piglets were pre-bled, then bled at 6 h and again at various days following injection (Fig. 5a).

Co-administration of mRNA-LNP encoding VNA-TcdB and EfAb to piglets resulted in detectable serum VNA-TcdB levels 6 h post injection, reached a peak at day 1, and remained at peak levels to day 5 (Fig. 5b). Intriguingly, co-administration of control mRNA-LNP in place of mRNA-LNP encoding-EfAb resulted in tenfold lower peak serum VNA-TcdB levels (Fig. 5b). With EfAb co-expressed, levels for both serum VNA and EfAb dropped steadily after day 5, displaying a half-life of about a week for both VNAs (Fig. 5b, VNA-TcdB represented by the lime colored plot), and EfAb (Fig. 5c). One additional piglet was injected with 1 mg/kg of mRNA-LNP encoding only VNA-TcdB (Fig. 5b). As expected, the increased dose of VNA-TcdB encoding mRNA-LNP led to higher serum VNA-TcdB levels (Fig. 5b, pink line). Nonetheless, even with a fivefold higher dose of RNA-encoded VNA-TcdB, the peak serum levels of VNA-TcdB were much lower than in the piglets in which EfAb encoding mRNA-LNPs was co-administered. Furthermore, in the absence of co-administered EfAb, VNA-TcdB serum levels also declined much faster. All piglet sera samples were monitored by ELISA for ADA (Supplement Fig. 9c) and no significant signs of an ADA response were detected to either VNA or EfAb (Supplement Fig. 9a,b).

In summary, co-administered EfAb substantially enhances the serum abundance and half-life of VNA-TcdB in the porcine model. Thus, a de-immunized two-component system should provide a viable approach to substantially improve the serum half-life of otherwise short-lived proteins such as VNAs.

## Discussion

In the current study we evaluated the therapeutic potential of mRNA-encoded heterodimeric VNAs that target each of the two major *C. difficile* virulence factors, TcdA and TcdB, and an mRNA-encoded heterotetrameric VNA that targets both toxins. The V_H_H heterotetramer (VNA-TcdA/B) was previously shown to protect from *C. difficile* infection pathology in three different animal models^5^. mRNA encoding the three VNAs were transfected to cells and their in vitro target binding and neutralization activity was established. Subsequently, mRNA encoding the VNAs were encapsulated into mRNA-LNPs, administered to mice, and the protective efficacy of the expressed VNAs was confirmed by improved clinical outcomes following toxin challenges.

VNAs are characterized by a short serum half-life^18^. In an effort to improve their therapeutic window, we tested the half-life extending potential of co-treatment with an anti-tag effector antibody (EfAb) binding corresponding tags on the VNAs^13, 14^ without altering their functionality and therapeutic potential against CDI. We employed an EfAb targeting O-tag epitopes (derived from^40^) which were included in the RNA-expressed VNAs. We demonstrated the effectiveness of this concept in both mice and piglets by co-administration of EfAbs with mRNA-LNPs encoding heteromultimeric V_H_H-based agents. In mice, we co-administered EfAb either as either recombinant protein or encoded in a humanized form by mRNA-LNP. When mRNA-LNP-encoded EfAb and VNA were co-administered to either mice or piglets at doses resulting in similar molar levels, VNA serum half-life was extended to equal the EfAb’s half-life. The improved serum VNA half-life prolonged antitoxin efficacy for several weeks in mice and increased peak serum VNA levels about tenfold in piglets. These results indicate that VNAs can adopt the Fc mediated enhanced serum half-life of a co-administered EfAb by forming an in vivo VNA:EfAb complex, mediated in this study by the O-tag epitopes. We therefore expect that VNAs in these EfAb complexes should also adopt other Fc effector functions such as pathogen opsonization or ADCC enhancing their therapeutic efficacy in other applications.

A thorough immunological analysis revealed that ADA was a complicating factor in the murine model, affecting serum titers of both components (VNA and EfAb), and consequently hampered protective efficacy in the affected animals. Interestingly, if mounted, the ADA response was directed to both components (VNA and EfAb) which might be due to increased immunogenicity of the VNA:EfAb complex. In a previous study, two different monoclonal antibodies (CR8033^43^ and SO57^42^) were administered as mRNA-LNPs to immunocompetent mice. While mRNA-LNP-encoded CR8033 remained unaffected for several weeks, SO57 induced ADA in a significant proportion of the animals^37^. Intriguingly, we did not observe evidence of ADA development to human mAbs after three weeks in non-human primates (Supplement Fig. 10b). Likewise, we did not find any accelerated decline of the human EfAb levels or associated ADA over two weeks in the porcine model. However, a greater number of piglets would be required to rule out the possibility of ADA with higher certainty. Overall, those findings emphasize that reducing host-specific immunogenicity and choosing a host closer to human condition is crucial for long-term animal studies.

For EfAbs, reducing immunogenicity might simply require the use of IgGs that are modified to employ Fc sequences that are based on IgGs from the species employed in the study. Such modifications have long been employed to reduce immunogenicity of IgG therapeutics for both humans^44, 45^ and veterinary animals^46^. Camelid V_H_Hs such as the components of VNAs in this study can also be modified to reduce immunogenicity^47^ and therapeutics employing humanized V_H_Hs are being commercialized^48^.

Taken together, we present evidence that the inherently short serum half-life of VNAs can be substantially improved by co-administration of an EfAb. While the current analysis focused on half-life prolongation, an EfAb might also transfer alternative Fc mediated functionalities to a target molecule^49^ and thus address other medical needs. For example, a different concept previously showed that in vivo antitoxin potencies of scFvs and VNAs can be enhanced, presumably through low affinity Fc-mediated clearance of the toxin once it becomes decorated with three or more Fc domains^50, 51^. The standard approach to adding Fc functions to V_H_H-based agents is to prepare these agents fused to Fc domains, although the Fc fusion partner can complicate expression challenges, particularly in microbial hosts^52–54^. While simple Fc fusions to V_H_H agents may be appropriate in some cases, we believe the two component EfAb option, despite the added complexity, offers several potential commercial advantages. In a two-component system, the framework of the target binding component (VNA) would be simple, rapid to develop and inexpensive to produce. Thus the tagged VNA component could be quickly developed and manufactured in response to new biothreats or for newly identified therapeutic targets^55^. Meanwhile the EfAb component, perhaps containing Fc mutations optimized for specific effector functions of therapeutic importance, could be pre-manufactured and stockpiled. VNAs could be quickly screened in combination with different EfAbs for optimal therapeutic efficacy. Similarly, the two-component system should also be ideal in rapid high-throughput screening approaches in development of antibody therapeutics^56^. Whether the EfAb should be encoded by mRNA or produced as a recombinant protein remains to be determined. The challenges and opportunities of mRNA-LNP encoded antibodies have been discussed in recent review articles^38, 39^ highlighting that, like any new technology, mRNA must prove its value for protein replacement use. The presented preclinical data shows that mRNA is a powerful technology to encode various proteins like nanobodies or monoclonal antibodies and produces substantial serum protein levels even in larger animals. Because of the COVID-19 pandemic, appreciation of therapeutic mRNA has skyrocketed. Therefore, more than likely a lot more data on its therapeutic use is ahead.

## Materials and methods

### Protein design

VNA frameworks have been described previously^37^. In brief, VNAs contained a murine Ig kappa signal peptide (P01661, Uniprot), one epitopic O-tag (DELGPRLMGK), V_H_H domains targeting epitopes of *C. difficile* toxins A and B^11^, another O-tag and a carboxyl-terminal murine ABP^57^. VNAs lacking the ABP contained one E-tag epitope (GAPVPYPDPLEPR) and one HA-tag epitope (YPYDVPDYA). The O-tag epitope is a modified OLLAS tag^40^ in which several amino acids at the amino end of the original OLLAS tag were found to be unnecessary for strong mAb binding and eliminated. The recombinant rat OLLAS mAb (used as the EfAb in this study) has been reported previously^40^.

The design of the RNA-encoded EfAb was based on the variable regions of the rat EfAb^40^ fused to a human heavy chain IgG1 framework (IGHG1*03 / G1m17,1; K120; E12/M14; derived from P01857, Uniprot) and to a kappa light chain (P01834, Uniprot). The signal peptides for heavy and light chain were derived from P01750 (Uniprot) and Q6P5S8 (Uniprot), respectively. All sequences can be found in Supplement Table 1. The sequence of SO57 antibody has been described previously^37^. The non-translated version of GFP lacked a start codon and included several stop codons.

### RNA design

The design and synthesis of mRNA sequences have been described previously^58, 59^. In brief, mRNAs contained a 5’ UTR, the open reading frame, a 3’ UTR and a poly-A sequence followed by a C30 stretch and a histone stem loop and involved unmodified nucleotides. Following in vitro transcription, mRNAs were enzymatically capped and 2′-O-methylated using vaccinia virus capping and 2’-O-Methyltransferase enzymes (CELLSCRIPT) and enzymatically adenylated using A-Plus Poly(A) Polymerase (CELLSCRIPT). All mRNAs were purified by RP-HPLC as described previously^59^. A detailed protocol has been described elsewhere^60^.

### RNA formulation

In general, lipid nanoparticle formulation was performed with Acuitas therapeutics LNP technology and has been described previously^61^. In brief, the LNPs were generally composed of an ionizable amino lipid, phospholipid, cholesterol, and a PEGylated lipid. mRNAs encoding IgG were mixed at a molar ratio of 1.2:1 (HC:LC, EfAB) or 1.5:1 (HC:LC, SO57) before LNP formulation. For injections, mRNA-LNP was diluted in phosphate-buffered saline pH 7.4 (PBS). mRNAs-LNP encoding VNA-TcdB lacking ABP contained SM-102 as ionizable amino lipid.

### Cell transfections

Transfection of mRNAs into cells has been described previously^37^. In brief, baby hamster kidney cells (BHK, ATCC) were cultivated, transfected, and maintained in conditioned medium (RPMI 1640, 10% FCS, 1% Penicillin / Streptomycin, 1% L-Glutamine, Lonza). Supernatants were harvested and cell lysates were prepared at either 24 or 48 h post transfection.

### Protein purification

The production and purification of recombinant proteins has been described previously^62^.

### Western blot analysis

Cell lysates and supernatants were loaded on 12% criterion TGX gels according to manufacturer’s instructions (Bio-Rad). Following SDS-PAGE, samples were transferred onto a 0.22 µm nitrocellulose membrane using the criterion blotting system (Bio-Rad). All washing and incubation steps have been described previously^37^. For detection of O-tagged VNAs, the following antibodies have been used: OLLAS Epitope Tag Antibody (L2) from rat (Novusbio, 1:1,000) and a Rabbit anti-α/β tubulin (New England Biolabs, 1:1000) as primary antibodies, and Goat anti-Rat IgG (H + L) IRDye 800 CW (LI-COR, 1:15,000) as well as a Goat anti-Rabbit IgG (H + L) IRDye 680 RD (LI-COR, 1:15,000) as secondary antibodies. Primary antibodies were incubated for two hours. Secondary antibodies were incubated for one hour. For detection of RNA-encoded EfAb the following antibodies have been used: A Rabbit anti-tubulin UNLB (Cell Signaling Technology, 1:15,000) as primary antibody, a Goat anti-human IgG CW800 (LI-COR, 1:15,000) as well as a Goat anti-rabbit RD680 (LI-COR, 1:15,000) as secondary antibodies. Primary and secondary antibodies have been incubated for one hour. Protein detection and image processing were carried out in an Odyssey CLx^®^ Imaging system and Image Studio version 5.2.5 (LI-COR) according to manufacturer’s recommendations.

### ELISA

The general procedure of sandwich ELISA based detection and has been described previously^5^. In brief, all ELISAs were performed using serial fivefold dilutions of supernatants, sera, and standards. Microplates were coated overnight at 4°C and blocked for a period of one to two hours. Diluted samples as well as purified and quantified standards were incubated at room temperature for one hour followed by incubation of detection Abs for one hour. Plates were washed three times in between incubation steps. Finally, 3,3=,5,5=-tetramethylbenzidine (TMB, Sigma) was added, and reactions were terminated with 1M sulfuric acid at times optimized for each condition. Plates were measured at 450 nm with 570 nm correction in a BioTek Synergy plate reader. Antibodies for detection of VNAs, EfAb and ADA are summarized in Supplement Table S2. Serum VNA concentration quantification was performed as previously described^18^.The quantification of human IgG1 (RNA-EfAb) from cell culture supernatants has been described previously^37^. The quantification of VNA-TcdB lacking ABP was performed according to^37^ and involved a Rabbit anti-E-tag antibody for capture (Bethyl, 1:500) and a biotinylated Rabbit anti-HA-tag antibody (Abcam, 1:15,000) for detection.

To quantify human IgG1 (SO57) from *M. fascicularis* serum, a human therapeutic IgG1 ELISA kit (Cayman) was used according to manufacturer’s instructions. To measure the binding affinity of murine ABP to albumin, ELISA plates were either coated with murine or porcine albumin and serial dilutions of recombinant VNA-BoNTA containing an E-tag epitope and a carboxyl terminal murine ABP were added. An anti-E-tag antibody conjugated to horseradish peroxidase was used for detection.

### In vitro neutralization assays

The procedure has been reported previously^5, 17^. In brief, Vero cells (ATCC) were incubated with serial dilutions of conditioned medium from BHK cells, untransfected contols or transfected with mRNA encoding VNAs, and toxins were added to all wells for a period of 24 h. A blinded researcher assessed the percentage of cells that were rounded in each well, and this estimate was plotted as a function of the dilution of conditioned medium present within each well. .

### Animal experiments

Treatment and care of all animals used in experiments followed institutional animal care and use committee guidelines. Mouse and piglet studies were conducted at Department of Infectious Disease and Global Health, Tufts Cummings School of Veterinary Medicine (North Grafton, USA). All protocols describing animal use were approved by the Tufts University Institutional Animal Care Use Committee in accordance with the Guide for the Care and Use of Laboratory Animals of the National Research Council, USA. Six- to eight-week-old female outbred CD-1 mice (Charles River Labs, Wilmington, USA) were randomized based on body weight and organized in five mice per group or cohort. All injections were done intravenously in a volume of 100 µl into the tail vein. For dose finding studies, groups of mice received single intravenous injections of serially diluted mRNA-LNPs. Blood was harvested at 24 h post injection by either submandibular or retro-orbital bleeding and serum was prepared. In PK studies, for each time point, a separate cohort of mice was used. For co-administration of different mRNA-LNPs, a mixture of diluted mRNA-LNPs was prepared prior to co-injection. For co-administration of mRNA-LNP and recombinant EfAb, both components were pre-diluted in PBS to an intermediate twofold concentration, and mixed 1:1 prior intravenous co-injection.

Toxins were administered intraperitoneally at either 25 ng (early challenge, 24 h post mRNA-LNP treatment) or 50 ng (late challenge, 14 days post mRNA-LNP treatment) per mouse. Following intoxication, mice were monitored for signs and symptoms of toxemia (including lethargy, depression, anorexia, dehydration, ruffled coat, and hunched posture) to determine the clinical score (Supplement Table 3). Mice exhibiting signs of severe illness (lethargy, difficulty with ambulation, lack of responsiveness to tactile stimulation, wasting) or weight loss ≥ 15% body weight were humanely euthanized according to IACUC recommendations.

Gnotobiotic piglets were derived via caesarean section and maintained in sterile isolators for the duration of the experiment^63^. In general, groups of two piglets were used if not stated differently. mRNA-LNPs were injected intravenously at a total dose of 1 mg/kg into the jugular veins. For co-administration of different mRNA-LNPs, a mixture of diluted mRNA-LNPs was prepared prior to injection. Mixtures either contained mRNA-LNPs encoding VNA-TcdB together with mRNA-LNPs encoding EfAb or together with mRNA-LNPs containing a non-coding RNA (non-translated GFP). Blood was harvested by routine methods and serum was prepared.

PK studies involving VNA-TcdB lacking ABP were conducted at Preclinics GmbH (Potsdam, Germany). Six- to eight-week-old female Balb/c mice were injected via the tail vein (100 µl) and blood was obtained by retro-orbital bleeding. The reference of the animal welfare approval is 2347-14-2018 Ä 5 approved by the animal welfare commission of the State of Brandenburg (LAVG—Landesamt für Arbeitsschutz, Verbraucherschutz und Gesundheit). The study protocol was reviewed by the internal council of Preclinics GmbH.

Cynomolgus monkeys (2.5–3 years and 2.5–3.1 kg) were housed and experiments were performed at Envigo CRS, S.A.U (Barcelona, Spain; now part of Covance). Animals received a single bolus injection of LNP-formulated mRNA (0.2 or 1 mg/kg) encoding SO57 in phosphate buffered saline pH 7.4. The total volume for intravenous injections was 1 ml/kg. Blood was collected from the femoral vein and serum was prepared. The study was approved by local authorities and the reference number was WS50NL.

All animal studies complied with the ARRIVE guidelines.

#### Computational and statistical analysis

Data were analyzed and plotted using GraphPad Prism software version 9.3.1. Serum titers of O-tagged VNAs, recombinant and RNA-encoded EfAb in mice and piglets were generated by EC50 quantification using BioTek Gen5 software. Serum titers of VNAs containing an HA-/E-tag combination in mice as well as serum titers of SO57 antibody in *M. fascicularis* were calculated by non-linear regression analysis using Graphpad Prism software version 9.3.1. Serum titers were either depicted as whisker plots showing min to max levels or as connected curves with error bars representing the SD of the mean. Graphs showing OD levels [A450] of in vitro expressed VNAs show superimposed symbols (mean with SD) with connecting lines. Data associated with the analysis of ADA are represented as OD levels [A450] of individual animals. Clinical scores of challenged animals are represented as curves with error bars representing the SEM of the mean. In vitro expression data of RNA-encoded EfAb are depicted as floating bars showing min to max values. Correlation analysis of titers and ADA in mice was done using simple linear regression.

The statistical significance of differences between groups when comparing titers was examined using an ordinary one-way ANOVA followed by Šídák’s multiple comparison post test (Graphpad Prism software version 9.3.1). All titer data were log10-*t*ransformed for the statistical analysis. Each *P* value was multiplicity adjusted to account for multiple comparisons. Differences were considered significant at adjusted *P* values of < 0.05.

### Supplementary Information

Supplementary Information.

## Data Availability

Data supporting the findings of this study are available in this paper, Supplementary material, or are available from the corresponding author upon request.
