# Supplementary material for: Co-administration of an effector antibody enhances the half-life and therapeutic potential of RNA-encoded nanobodies
Source: Sci Rep. 2023 Sep 5;13:14632. doi: 10.1038/s41598-023-41092-7 (PMC10480410; doi:10.1038/s41598-023-41092-7)
Supplement: Supplementary file 1 — Supplementary Information. [file 41598_2023_41092_MOESM1_ESM.pdf]

Protein	Mass (w/o SP)	AA sequence (including SP)
VNA-TcdA	37 kDa	METDTLLLWVLLLWVPGSTGDAAQPARRARRTKLSGGGGDELGPRLMGKGGGGQGVQSQLQLVESGGGLVQPGGSLRLSCAASGFTLDYSSIGWFRQAPGKEREGVSCISSSGDSTKYADSVKGRFTTSRDNAKNTVYLQMNSLKPDDTAVYYCAAFRATMCGVFPLSPYGKDDWGKGTLVTVSSEPKTPKPQGGGGSGGGGSGGGGSQGVQSQVQLVESGGGLVQPGGSLRLSCAASGFTFSDYVMTWVRQAPGKGPEWIATINTDGSTMRDDSTKGRFTISRDNAKNTLYLQMTSLKPEDTALYYCARGRVISASAIRGAVRGPGTQVTVSSEPKTPKPQGGGGDELGPRLMGKGGGGSDICLPRWGCLWED
VNA-TcdB	36 kDa	METDTLLLWVLLLWVPGSTGDAAQPARRARRTKLSGGGGDELGPRLMGKGGGGQGVQSQLQLVESGGGLVQPGGSLRLSCEASGFTLDYYGIGWFRQPPGKEREAVSYISASARTILYADSVKGRFTISRDNAKNAVYLQMNSLKREDTAVYYCARRRFSASSVNRWLADDYDVWGRGTQVAVSSEPKTPKPQGGGGSGGGGSGGGGSQGVQSQVQLVESGGGLVQTGGSLRLSCASSGSIAGFETVTWSRQAPGKSLQWVASMTKTNNEIYSDSVKGRFIISRDNAKNTVYLQMNSLKPEDTGVYFCKGPELRGQGIQVTVSSEPKTPKPQGGGGDELGPRLMGKGGGGSDICLPRWGCLWED
VNA-TcdA/B	67 kDa	METDTLLLWVLLLWVPGSTGDAAQPARRARRTKLSGGGGDELGPRLMGKGGGGQGVQSQLQLVESGGGLVQPGGSLRLSCAASGFTLDYSSIGWFRQAPGKEREGVSCISSSGDSTKYADSVKGRFTTSRDNAKNTVYLQMNSLKPDDTAVYYCAAFRATMCGVFPLSPYGKDDWGKGTLVTVSSEPKTPKPQGGGGSGGGGSGGGGSQGVQSQLQLVESGGGLVQPGGSLRLSCEASGFTLDYYGIGWFRQPPGKEREAVSYISASARTILYADSVKGRFTISRDNAKNAVYLQMNSLKREDTAVYYCARRRFSASSVNRWLADDYDVWGRGTQVAVSSEPKTPKPQGGGGSGGGGSGGGGSQGVQSQVQLVESGGGLVQTGGSLRLSCASSGSIAGFETVTWSRQAPGKSLQWVASMTKTNNEIYSDSVKGRFIISRDNAKNTVYLQMNSLKPEDTGVYFCKGPELRGQGIQVTVSSEPKTPKPQGGGGSGGGGSGGGGSQGVQSQVQLVESGGGLVQPGGSLRLSCAASGFTFSDYVMTWVRQAPGKGPEWIATINTDGSTMRDDSTKGRFTISRDNAKNTLYLQMTSLKPEDTALYYCARGRVISASAIRGAVRGPGTQVTVSSEPKTPKPQGGGGDELGPRLMGKGGGGSDICLPRWGCLWED
VNA-BoNTA	35 kDa	METDTLLLWVLLLWVPGSTGDAAQPARRARRTKLSGGGGDELGPRLMGKGGGGQGVQAQLQLVESGGGLVQVGGSLRLSCVVSGSDISGIAMGWYRQAPGKRREMVADIFSGGSTDYAGSVKGRFTISRDNAKKTSYLQMNNVKPEDTGVYYCRLYGSGDYWGQGTQVTVSSAHHSEDPSGGGGSGGGGSGGGGSGGQGVQAQLQLVESGGGLVHPGGSLRLSCAPSASLPSTPFNPFNNMVGWYRQAPGKQREMVASIGLRINYADSVKGRFTISRDNAKNTVDLQMDSLRPEDSATYYCHIEYTHYWGKGTLVTVSSEPKTPKPQSGGGGDELGPRLMGKGGGGSDICLPRWGCLWED
RNA-EfAb heavy chain, human IgG1	49 kDa	MGWSCIILFLVATATGVHSEVQLVETGGGLVQPGRSLKLTCAASGFTFSAAWMHWVRQSPNKRLDWVARIKDKSNNYATDYVESVKGRFTISRDDSKSSLYLQMNNLKEEDSATYYCVTSEDWGQGVMVTVSSASTKGPSVFPLAPSSKSTSGGTAALGCLVKDYFPEPVTVSWNSGALTSGVHTFPAVLQSSGLYSLSSVVTVPSSSLGTQTYICNVNHKPSNTKVDKRVEPKSCDKTHTCPPCPAPELLGGPSVFLFPPKPKDTLMISRTPEVTCVVVDVSHEDPEVKFNWYVDGVEVHNAKTKPREEQYNSTYRVVSVLTVLHQDWLNGKEYKCKVSNKALPAPIEKTISKAKGQPREPQVYTLPPSREEMTKNQVSLTCLVKGFYPSDIAVEWESNGQPENNYKTTPPVLDSDGSFFLYSKLTVDKSRWQQGNVFSCSVMHEALHNHYTQKSLSLSPGK
RNA-EfAb light chain, kappa	24 kDa	METPAQLLFLLLLWLPDTTGDIQMTQSPSFLSASVGDRATFNCKASQNIARSLNWYQQKLGEAPKLLIYNTNTLQPGIPSRFSGSGSGTDFTLTISSLQPEDVATFFCLQFGSWPLTFGSGTKLEIKRRTVAAPSVFIFPPSDEQLKSGTASVVCLLNNFYPREAKVQWKVDNALQSGNSQESVTEQDSKDSTYSLSSTLTLSKADYEKHKVYACEVTHQGLSSPVTKSFNRGEC

Supplement table 1: Information on protein sequences
Summary of proteins encoded by mRNA used for this publication. Molecular mass is depicted as average mass excluding the mass of signal peptides.
VNA sequences include a signal peptide, an O-tag, V_HH sequences, a second O-tag, and the ABP. EfAb sequences include a signal peptide, variable, and constant regions.

Method reference	Capture agent (Source, concentration)	Samples	Detection agent (Species, source, dilution)	Used to detect
O-Tag detection ELISA	Tcd toxin A or B (In house, 1 µg/ml)	BHK supernatant	Anti-O-tag HRP Ab (Rat, in house, 1 ug/ml)	Free O-tagged VNA (Non-complexed)
		Murine serum		
V_HH detection ELISA	Tcd toxin (In house 0.5 µg/ml)	Murine serum	Anti-V_HH rabbit serum (In house) + Anti-Rabbit HRP (Goat, Southern Biotech, 1:1,000)	Total VNA-TcdB (Non-complexed + complexed)
		Porcine serum		
Rat EfAb ELISA	VNA-TcdA-O-Tag (In house, 10 ug/ml)	Murine serum	Anti-rat HRP (Goat, Santa Cruz, 1:2,000)	Rat recombinant EfAb (Non-complexed)
Total human IgG ELISA	Anti-human IgG (ThermoFisher kit, as recommended)	Murine serum	Anti-human IgG HRP (Donkey, Jackson Labs, 1:5,000)	Total human IgG (RNA-EfAb)
	Anti-human Fc V_HH (In house (JYN-B4), 1 µg/ml)	Porcine serum		
VNA ADA ELISA	VNA-TcdB (E-tag) (In house, 1 ug/ml)	Murine serum	Anti-mouse IgG HRP (Goat, Santa Cruz, 1:2,000)	Anti-VNA ADA
		Porcine serum	Anti-pig IgG HRP (Goat, Pierce, 1:2,000)	
Murine EfAb ADA ELISA	Anti-Stx2 human IgG (In house (5C12), 2 ug/ml)	Murine serum	Anti-mouse IgG HRP (Goat, Santa Cruz, 1:2,000)	Anti-EfAb ADA
Porcine EfAb ADA ELISA	Human IgG (Anti-Ollas) (In house, 1 µg/ml)	Porcine serum	Anti-pig IgG HRP (Goat, Pierce, 1:2,000)	Anti-EfAb ADA

Supplement table 2: Summary of ELISA procedures
Summary of ELISA procedures for detection of individual VNAs, recombinant rat or mRNA-encoded human EfAb and detection of ADA responses.

Category	Severity	Abbreviation	Clinical score
Bloating (category 1)	Mild	MB	2
	Moderate	B	3
	Severe	SB	4
Breathing (category 2)	Slightly heavy	SHB	1
	Rapid heavy	RHB	2
	Labored	LB	3
	Severely labored	SLB	4
	Erratic (jumpy)	EB	4
Miscellaneous (categories 3-5)	Ruffled fur	RF	1
	Hunched posture	H	1
	Wobble	W	5

Category score ranging from 2-4

Category score ranging from 1-4

Category score = 1

Category score = 1

Category score = 5

Highest theoretical score = 15

Supplement table 3: Summary of animal scoring criteria

Summary of criteria for scoring animals based on different categories. The maximum theoretical clinical score an animal could reach was 15 (additive from each individual category). Due to IACUC guidelines, whenever possible, the animals were euthanized before progressing to higher clinical scores.

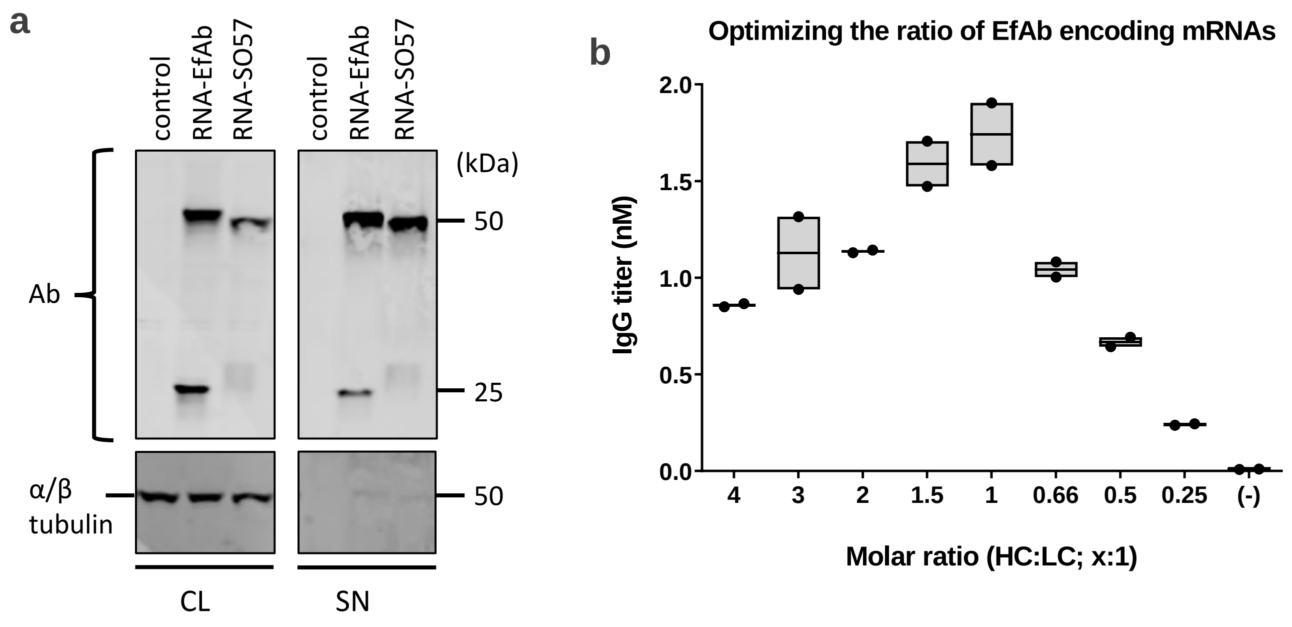

Supplement figure 1: ***In vitro*** **expression of RNA-EfAb**

a Western blot analysis of mRNA-encoded EfAb and SO57 in lysates and supernatants of transfected BHK cells, 48 hours post transfection. Samples were loaded on denaturing SDS-PAGE. Signals correspond to heavy and light chains (upper blot) and α/β tubulin as a loading control (lower blot). An uncropped version can be found in Supplement Fig. 12.

b Determination of the optimal molar ratio between heavy and light chain (HC:LC) encoding mRNAs in vitro. Quantification of mAb levels in supernatants of transfected BHK cells, 24 hours post transfection by IgG specific ELISA with different indicated molar ratios between heavy and light chain encoding mRNAs. Floating bars represent min to max values of duplicates. Untreated cells were used as control (-).

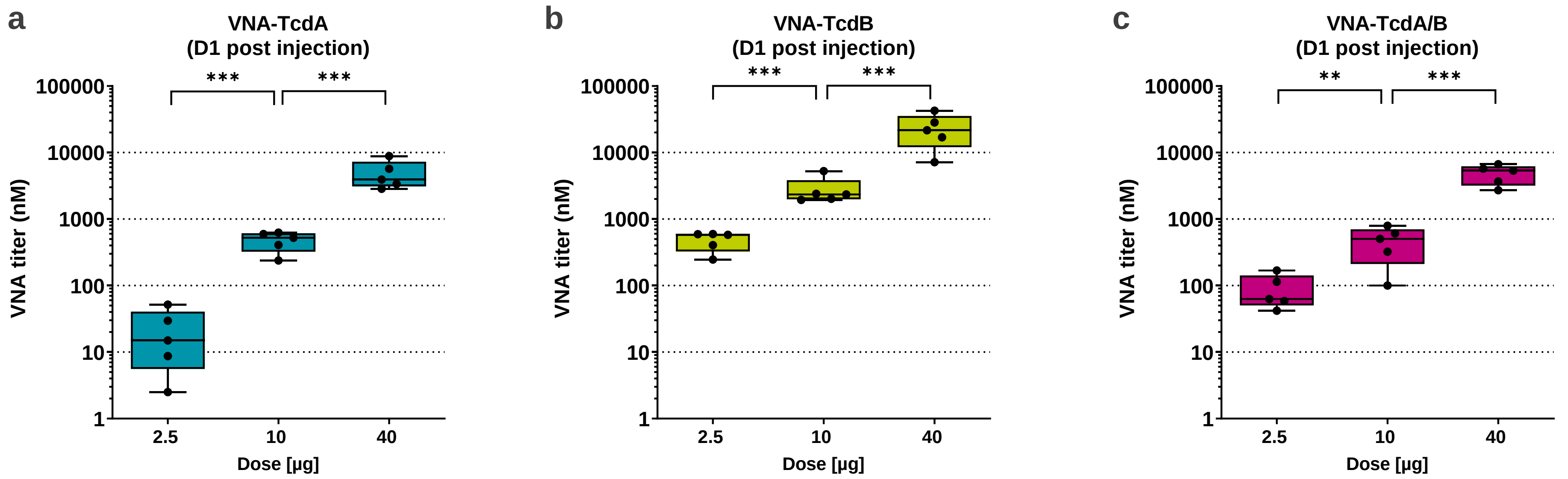

Supplement figure 2: ***In vivo*** **dose response for mRNA-LNP-encoded VNAs**

a-c Quantification of serum VNA titer (O-tag detection ELISA, Supplement table 2), one day following a single intravenous injection of various doses of mRNA-LNP encoding VNA-TcdA (a), VNA-TcdB (b) and VNA-TcdA/B (c) in outbred CD-1 mice. Data is depicted as whisker plots showing min to max values of five individual mice. $*p < 0.05$, $**p < 0.01$, $***p < 0.001$.

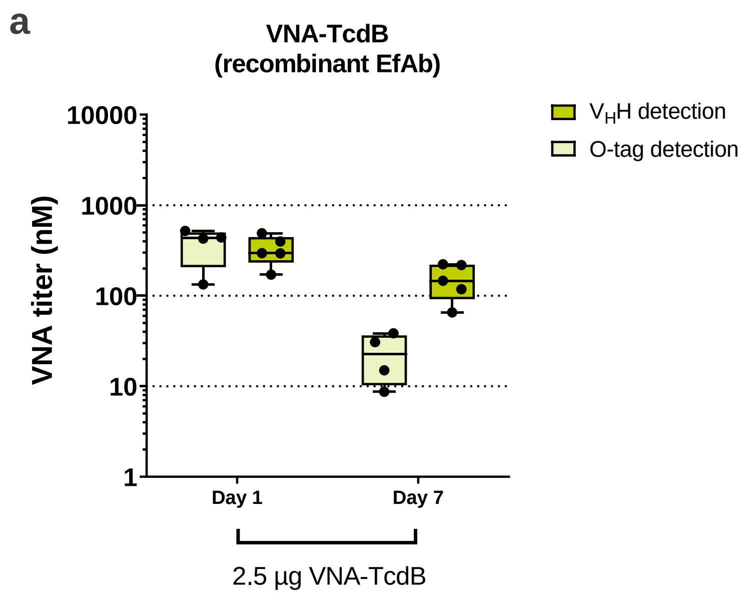

Supplement figure 3: Detecting the VNA half-life stabilizing effect of co-administered EfAb requires V_HH detection ELISA
Female outbred CD-1 mice received a single intravenous injection of 100 µg of recombinant EfAb co-administered with 2.5 µg of mRNA-LNP encoding VNA-TcdB. Quantification of serum VNA-TcdB titers was done by ELISAs either detecting the V_HH domain or the O-tags of VNA-TcdB (Described in Supplement table 2). Data is depicted as whisker plots showing min to max values of four or five individual mice.

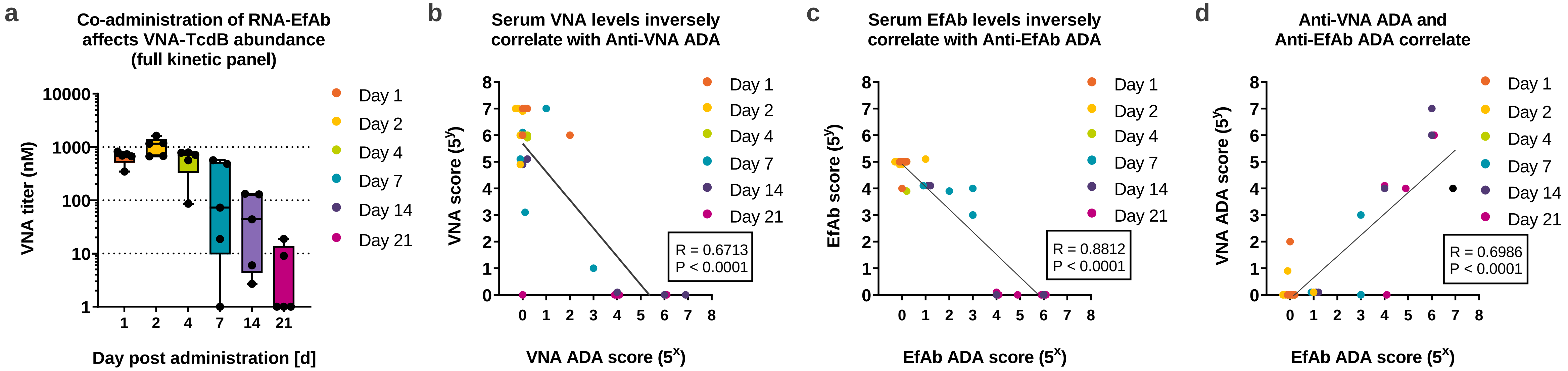

Supplement figure 4: ADA affects the PK of mRNA-LNP encoded VNA-TcdB

Correlation between serum levels and anti-drug antibodies in outbred CD-1 mice explains heterogenous VNA levels at later timepoints following injection of mRNA-LNP.

a Quantification of serum VNA-TcdB titer (V_HH detection ELISA, Supplement table 2; Same data points from day 1 and day 7 as shown in Fig. 3e) at various timepoints following a single intravenous injection of 2.5 µg of mRNA-LNP encoding VNA-TcdB together with 10 µg of mRNA-LNP-encoded EfAb in mice. Data is depicted as whisker plots showing min to max values of five individual mice. Colors indicate respective timepoints following injection.

b Correlation analysis of VNA titers and ADA interrogating heterogenous serum VNA-TcdB titers at later timepoints in a). VNA ADA score (determined in Supplement Figs. 5a,b) were plotted against VNA score (determined in Supplement Figs. 5c,d). Simple linear regression was used to analyze correlation. Colors indicate respective timepoints following injection.

c Correlation analysis of EfAb titers and ADA interrogating heterogenous serum VNA-TcdB titers at later timepoints in a). EfAb ADA score (determined in Supplement Figs. 6a,b) were plotted against EfAb score (determined in Supplement Figs. 6c,d). Simple linear regression was used to analyze correlation. Colors indicate respective timepoints following injection.

d Correlation analysis of VNA ADA and EfAb ADA. VNA ADA score (determined in Supplement Figs. 5a,b) were plotted against EfAb ADA score (determined in Supplement Figs. 6a,b). Simple linear regression was used to analyze correlation. Colors indicate respective timepoints following injection.

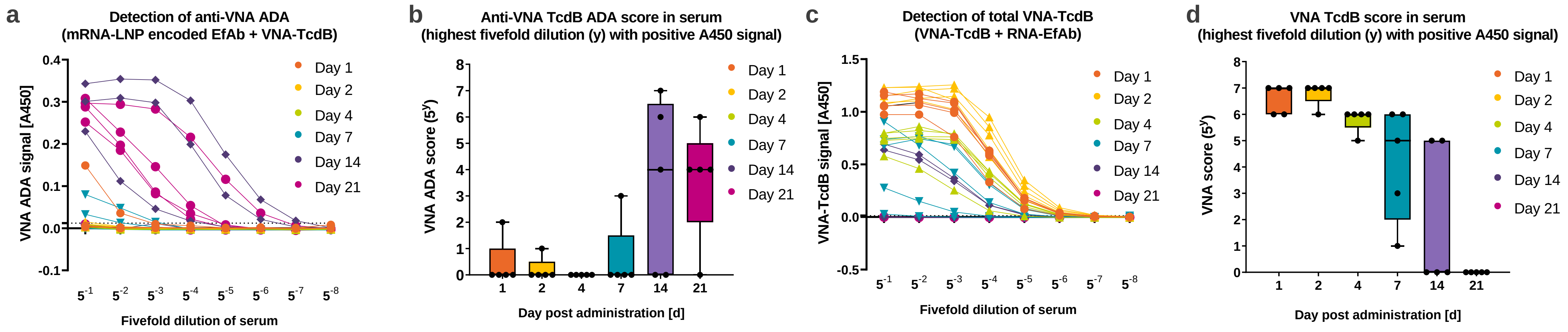

e

Method reference	Capture agent	Detection agent	Detection
VNA ADA ELISA	VNA-TcdB	Anti-mouse HRP	VNA-TcdB ADA
V_HH detection ELISA	TcdB toxin	Anti-V_HH serum / Anti-Rabbit HRP	Total VNA-TcdB

Supplement figure 5: Scoring of VNA and anti-VNA ADA signals

Scoring of VNA and anti-VNA ADA signals following a single intravenous injection of 2.5 µg of mRNA-LNP encoding VNA-TcdB together with 10 µg of mRNA-LNP encoding EfAb in outbred CD-1 mice (Experiment shown in Supplement Fig. 4a).

a Detection of anti-VNA-TcdB ADA in serum (VNA ADA ELISA). Sera were serially diluted (fivefold) and applied to ELISA plates. A450 values (y-axis) are plotted against the dilution (x-axis).

b VNA ADA scoring based on A450 values from a). A450 values above unspecific background were considered as positive signals. The highest dilution showing a positive signal for each sample was considered the score. The threshold was identical to the highest A450 signal for unspecific control samples (mock sera). The calculated and applied threshold depicted as dashed line was 0.012 (A450).

c Detection of total VNA-TcdB in serum (V_HH detection ELISA). Sera were serially diluted (fivefold) and applied to ELISA plates. A450 values (y-axis) are plotted against the dilution (x-axis).

d VNA scoring based on A450 values from c). A450 values above unspecific background were considered as positive signals. The highest dilution showing a positive signal for each sample was considered the score. The threshold was identical to the highest A450 signal for unspecific control samples (mock sera). The calculated and applied threshold depicted as dashed line was 0.007 (A450).

e Table summarizing details on ELISA procedures for a) and c).

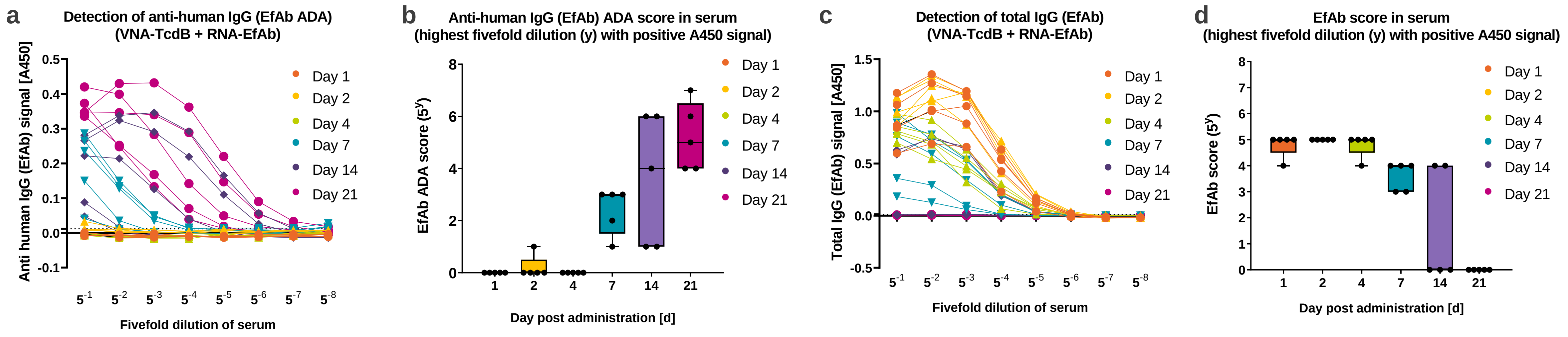

e

Method reference	Capture agent	Detection agent	Detection
Murine EfAb ADA ELISA	Human IgG Ab (Anti-Stx2)	Anti-mouse HRP	Anti-human IgG (EfAb ADA)
Total human IgG ELISA	Anti human IgG	Anti human IgG HRP	Total human IgG (EfAb)

Supplement figure 6: Scoring of EfAb and Anti-IgG (EfAb) ADA signals

Scoring of EfAb and Anti-IgG (EfAb) ADA signals following a single intravenous injection of 2.5 µg of mRNA-LNP encoding VNA-TcdB together with 10 µg of mRNA-LNP encoding EfAb in CD-1 mice (Experiment shown in Supplement Fig. 4a).

a Detection of anti-IgG (EfAb) ADA in serum (Murine EfAb ADA ELISA, Supplement table 2). Sera were serially diluted (fivefold) and applied to ELISA plates. A450 values (y-axis) are plotted against the dilution (x-axis).

b EfAb ADA scoring based on A450 values from a). A450 values above unspecific background were considered as positive signals. The highest dilution with a still positive signal for each sample was considered the score. The threshold was identical to the highest A450 signal for unspecific control samples (mock sera). The calculated and applied threshold depicted as dashed line was 0.02 (A450).

c Detection of EfAb in serum (Total human IgG ELISA, Supplement table 2). Sera were serially diluted (fivefold) and applied to ELISA plates. A450 values (y-axis) are plotted against the dilution (x-axis).

d EfAb scoring based on A450 values from c). A450 values above unspecific background were considered as positive signals. The highest dilution with a still positive signal for each sample was considered the score. The threshold was identical to the highest A450 signal for unspecific control samples (mock sera). The calculated and applied threshold depicted as dashed line was 0.054 (A450).

e Table summarizing details on ELISA procedures for a) and c).

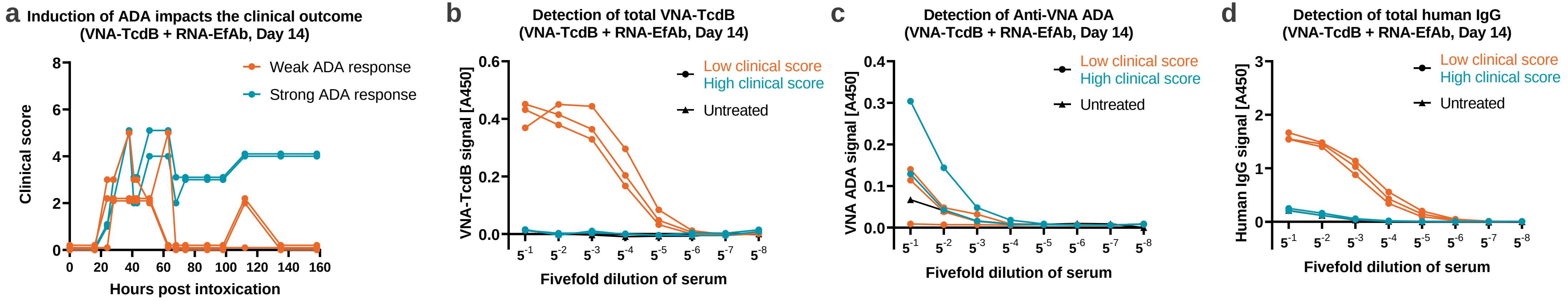

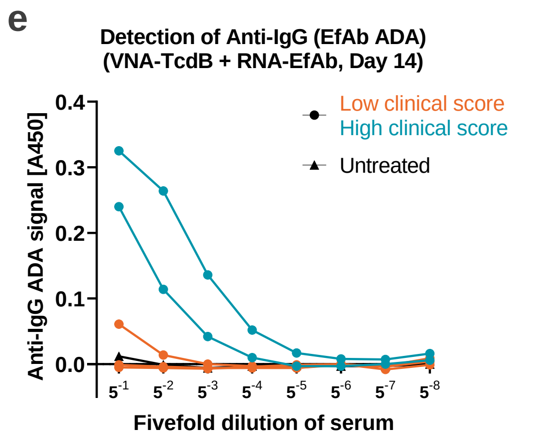

f

Method reference	Capture agent	Detection agent	Detection
Murine EfAb ADA ELISA	Human IgG Ab (Anti-Stx2)	Anti-mouse HRP	Anti-human IgG (EfAb ADA)
Total human IgG ELISA	Anti human IgG	Anti human IgG HRP	Total human IgG (EfAb)
VNA ADA ELISA	VNA-TcdB	Anti-mouse IgG HRP	Anti-VNA ADA
V_HH detection ELISA	TcdB toxin	Anti-V_HH serum / Anti-Rabbit HRP	Total VNA-TcdB

Supplement figure 7: Correlation of clinical outcome following a late challenge in outbred CD-1 mice

Mice which had received a single intravenous injection of 2.5 µg of mRNA-LNP encoding VNA-TcdB together with 10 µg of mRNA-LNP encoding EfAb (same animals as shown in Fig. 4c) were challenged after 14 days with 50 ng of TcdB toxin, and the clinical outcome was scored (Supplement table 3). Serum levels of VNA, EfAb, Anti-VNA-TcdB ADA and Anti-EfAb ADA at the time of challenge were measured by specific ELISAs.

a Individual clinical scores from mice injected with 2.5 µg of mRNA-LNP encoding VNA-TcdB together with 10 µg of mRNA-LNP encoding EfAb (extracted from Fig. 4c).

b Detection of VNA-TcdB in serum (V_HH detection ELISA). Sera were serially diluted (fivefold) and applied to ELISA plates. A450 values (y-axis) are plotted against the dilution (x-axis).

c Detection of Anti-VNA ADA (VNA ADA ELISA). Sera were serially diluted (fivefold) and applied to ELISA plates. A450 values (y-axis) are plotted against the dilution (x-axis).

d Detection of EfAb (Total human IgG ELISA). Sera were serially diluted (fivefold) and applied to ELISA plates. A450 values (y-axis) are plotted against the dilution (x-axis).

e Detection of Anti-IgG (EfAb) ADA in serum (Murine EfAb ADA ELISA). Sera were serially diluted (fivefold) and subjected to ELISA plates. A450 values (y-axis) are plotted against the dilution (x-axis).

f Table summarizing details on ELISA procedures for b-e.

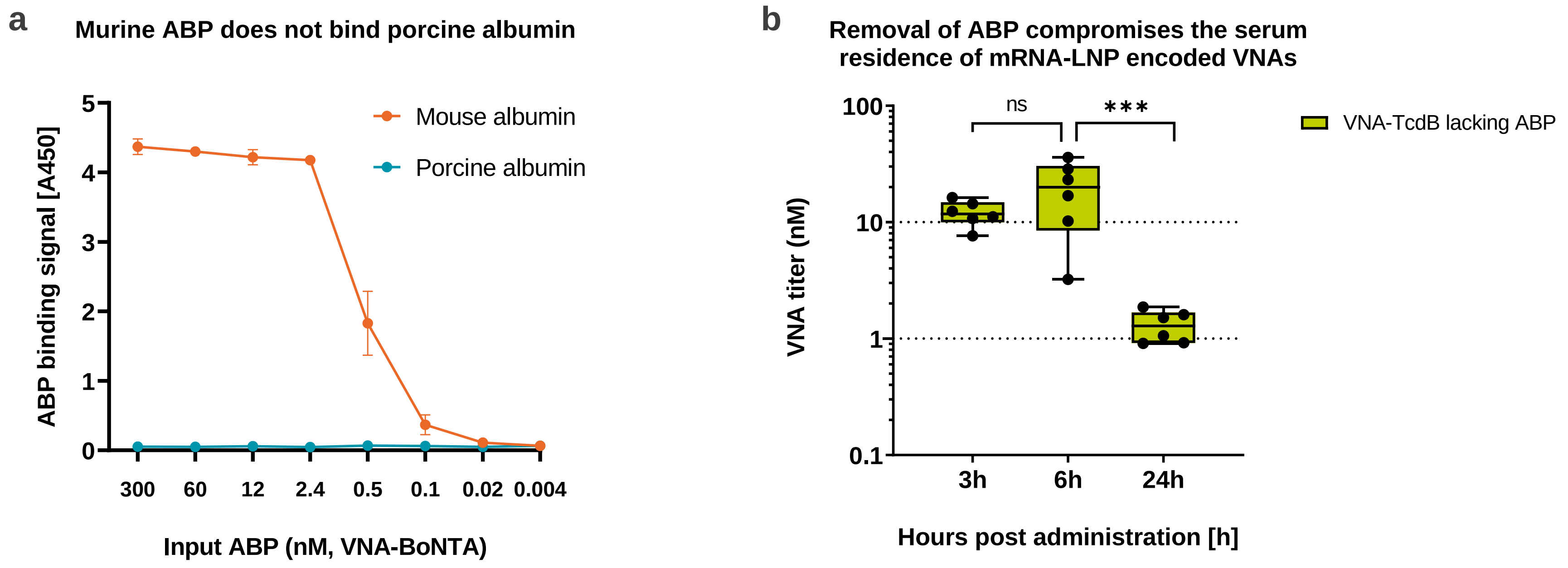

Supplement figure 8: Impact of ABP functionality on half-life of mRNA-LNP encoded VNAs

a Detection of ABP binding to porcine or murine albumin. ELISA plates coated with porcine or murine albumin were incubated with serial dilutions of murine ABP containing VNA-BoNTA. A450 values (y-axis) are plotted against the dilution (x-axis). Error bars represent the SD from four replicates.

b Serum VNA-TcdB levels in Balb/c mice which received a single intravenous injection of 10 µg of mRNA-LNP encoding and E-/HA-tagged VNA-TcdB lacking an ABP. Quantification was done by an ELISA specific for the E-/HA-tag combination. Data is depicted as whisker plots showing min to max values of six individual mice. *$p < 0.05$, **$p < 0.01$, ***$p < 0.001$.

a

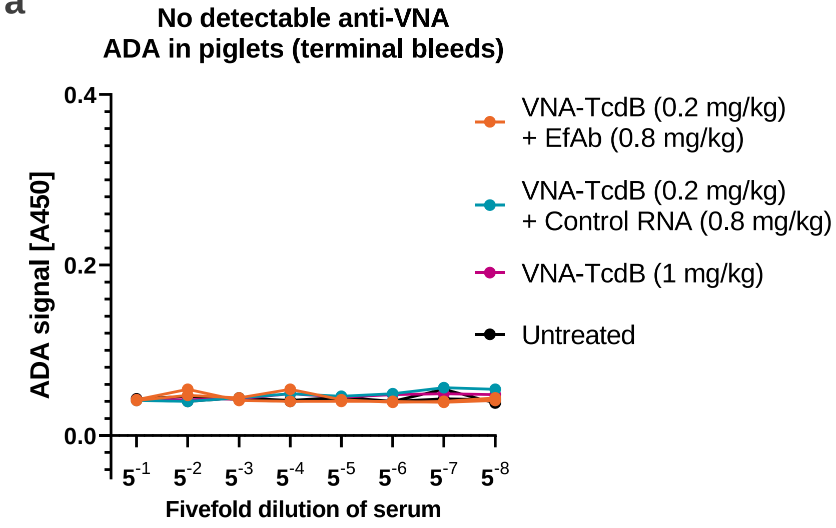

b

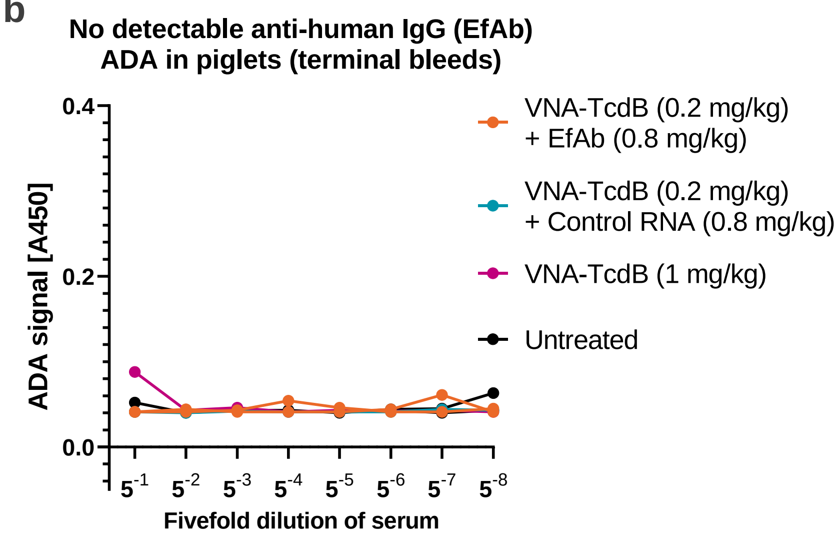

c

Method reference	Capture agent	Detection agent	Detection
VNA ADA ELISA	VNA-TcdB	Anti-pig IgG	Anti-VNA-TcdB ADA
Porcine EfAb ADA ELISA	Human IgG Ab	Anti-pig IgG	Anti-human IgG ADA

Supplement figure 9: No signs of ADA in piglets following administration of mRNA-LNP encoding VNA-TcdB and EfAb

a Detection of Anti-VNA ADA in porcine sera from the terminal bleeding (Fig. 5a) by VNA ADA ELISA. Data is depicted as lines representing the A450 value of individual animals derived from piglets from Figs 5b,c.

b Detection of Anti-EfAb ADA in porcine sera from the terminal bleeding (Fig. 5a) using porcine EfAb ADA ELISA. Data is depicted as lines representing the A450 value of individual animals derived from piglets from Figs 5b,c.

c Table summarizing details on ELISA procedures for a) and b).

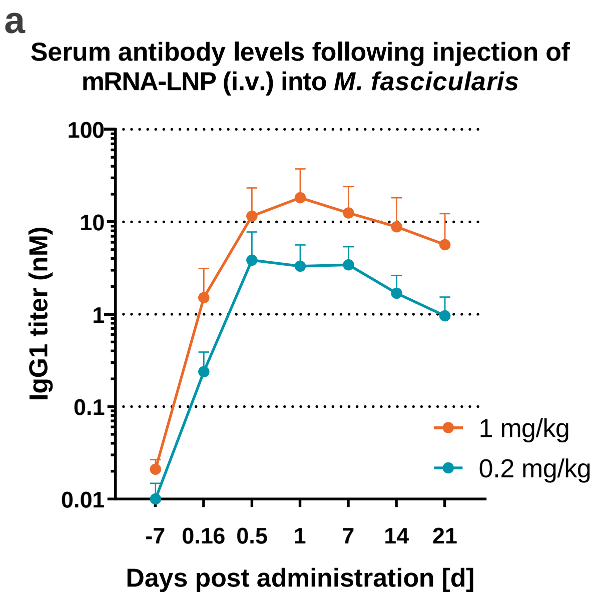

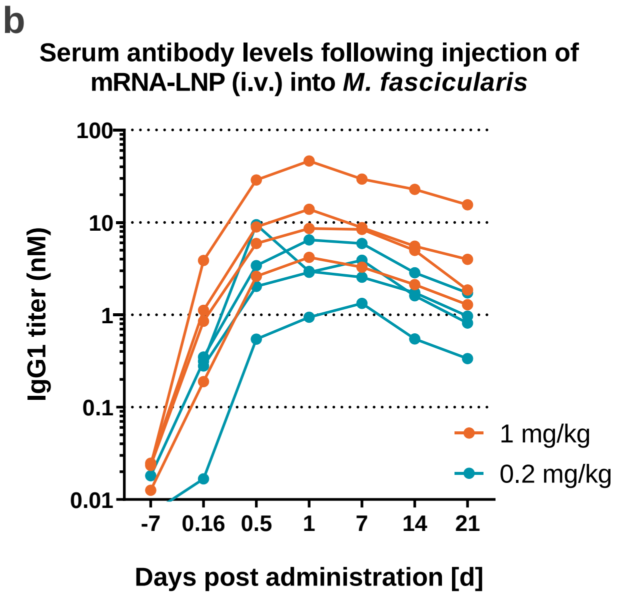

Supplement figure 10: Dose response and PK of mRNA-LNP encoded SO57 (human IgG1) in non-human primates

No signs of accelerated drop in antibody levels (as an indicator of ADA) were observed in the individual animals throughout the 3 weeks period.

a Dose response quantification of human IgG1 titer in macaque serum at various timepoints following a single intravenous injection of either 0.2 or 1 mg/kg of mRNA-LNP encoding SO57 mAb by ELISA specific for human IgG1. Data is depicted as mean. Error bars represent the SD of four animals.

b PK analysis by depicting human IgG1 levels from a) from individual macaque sera.

a

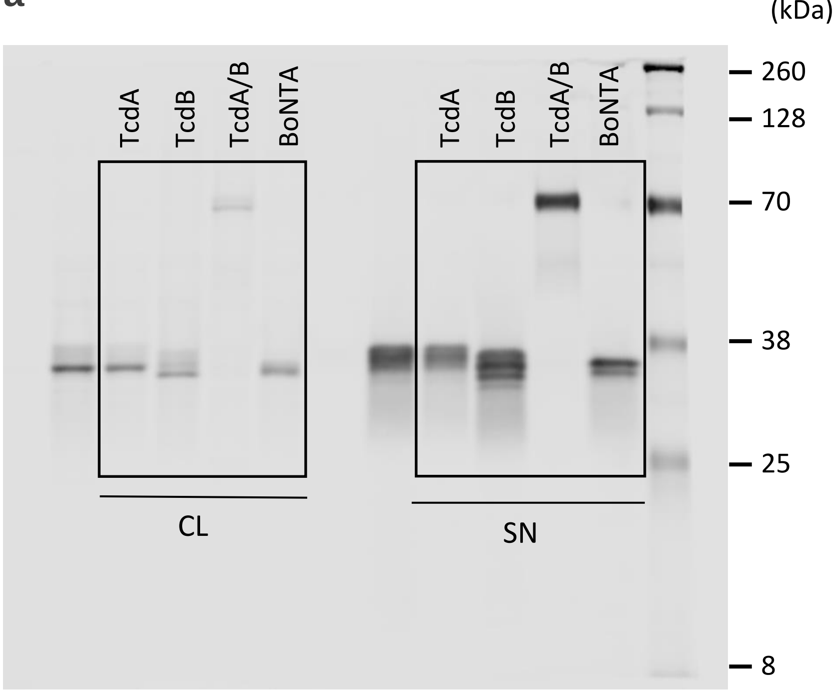

Channel 800/detection of VNA.
(Detection Ab binding to O-tag)
Irrelevant lanes were removed for Figure 1b.

b

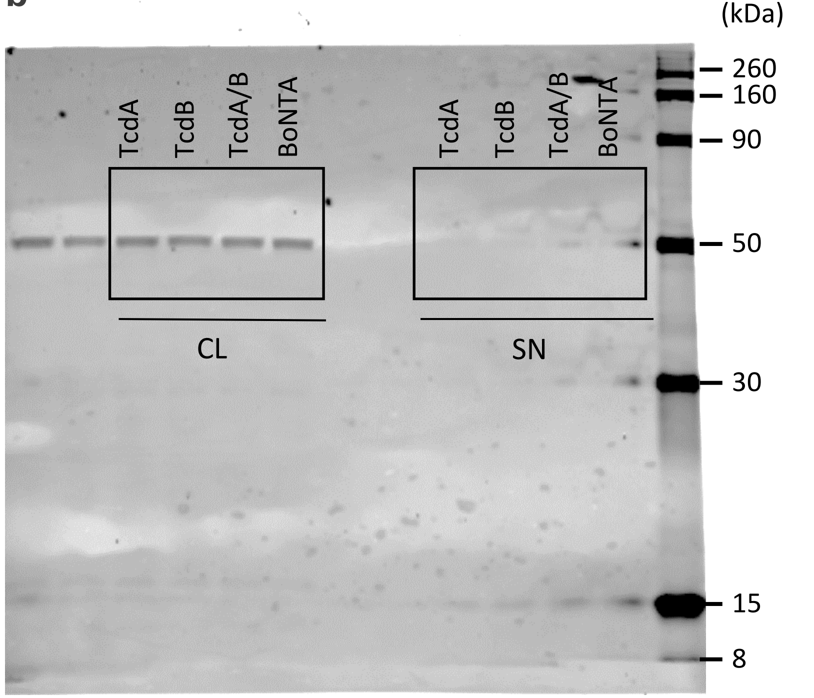

Channel 700/detection of α/β tubulin.
Irrelevant lanes were removed for Figure 1b.

Supplement figure 11: Original Western blot data for mRNA encoded VNAs
Uncropped version of the cropped Western blot images from Fig. 1b which shows the appearance of the relevant O-tagged VNAs used within the study. The same membrane was incubated with an antibody detecting the O-tags (a) contained in the expressed VNAs and an antibody detecting α/β tubulin (b). The blot image was cropped to remove irrelevant lanes.

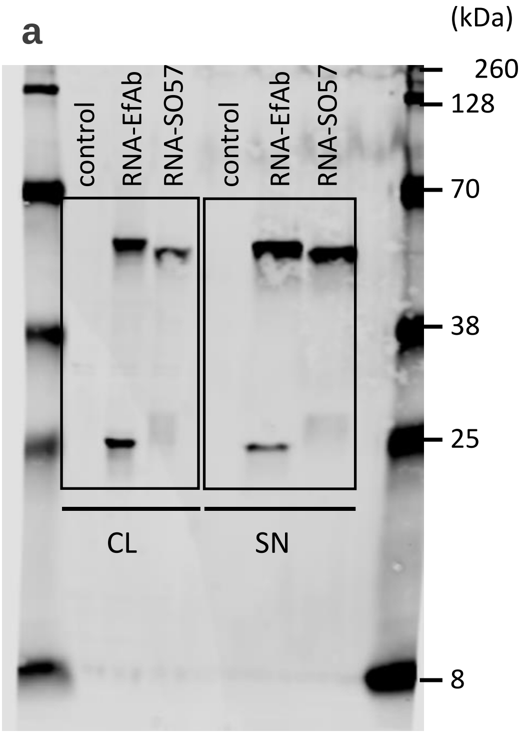

Channel 800/detection of EfAb.
(Detection Ab binding to human IgG heavy and light chain). Empty lanes were removed for Supplement Fig. S1.

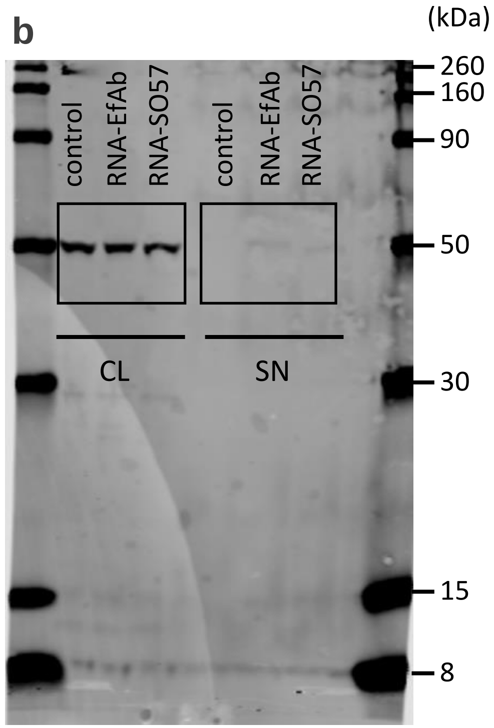

Channel 700/detection of α/β tubulin.
Empty lanes were removed for Supplement Fig. S1.

Supplement figure 12: Original Western blot data for mRNA encoded EfAb
Uncropped version of the cropped Western blot images from Supplement Fig. 1a which shows the appearance of EfAb used within the study.
The same membrane was incubated with an antibody detecting heavy and light chains of human IgG (a), contained in the EfAb and an antibody detecting α/β tubulin (b).
The blot image was cropped to remove empty lanes.
